# Sustainability indicators for salmon aquaculture

**DOI:** 10.1016/j.dib.2018.07.043

**Published:** 2018-07-27

**Authors:** Vilde Steiro Amundsen, Tonje Cecilie Osmundsen

**Affiliations:** a Department of Sociology and Political Science, Faculty of Social and Educational Sciences, NTNU - Norwegian University of Science and Technology/Studio Apertura, NTNU Social Research, Dragvoll allé 38 B, 7049 Trondheim, Norway; b Studio Apertura, NTNU Social Research, Dragvoll allé 38 B, 7049 Trondheim, Norway

## Abstract

In this paper, we present and describe data comprising indicators of sustainability, collected from eight of the major certification schemes for salmon aquaculture and categorized according to the topics covered by each. These indicators cover most aspects of aquaculture production, including biotic and abiotic effects, feed, emission and waste, fish health and welfare, social assurance, and respect for native culture. In addition to being published in its entirety as supplementary material alongside this article, the data is available through a searchable database on the SustainFish project site: https://sustainfish.wixsite.com/sustainfishproject/search-indicator-database.

**Specifications table**

**Table:** 

Subject area	*Political science, anthropology, marine social science, economics, marine biology*
More specific subject area	*Sustainability, salmon aquaculture*
Type of data	*Table, figure*
How data was acquired	*The data was obtained and categorized from certification scheme standards for (salmon) aquaculture*
Data format	*Raw and partially analyzed*
Experimental factors	*None*
Experimental features	*Qualitative data analysis*
Data source location	*Not Applicable*
Data accessibility	*Data is presented in this article and it is freely and publicly available for any academic, educational, and research purposes. Searchable database available at*https://sustainfish.wixsite.com/sustainfishproject/search-indicator-database

**Value of the data**

• The data gives an orderly overview of indicators used by certification schemes to regulate the salmon aquaculture industry.
• The data is comparable to former and future sets of indicators, allowing insight into the evolvement of focus areas in the regulation of aquaculture.
• The data serves as a foundation for researchers in developing new indicators.
• The data provides policy-makers and industry actors with an extensive and easily searchable collection of indicators applicable for improved regulation of the aquaculture industry.

## 1 Data

The indicators collected in this dataset are obtained from eight of the major certification schemes and their standards pertaining to salmon aquaculture (see Table 1). The aquaculture industry, with its incredible growth and countless challenges [1], [2], [3], [4], has seen a substantial increase in private regulatory agents such as these certification schemes. The recent surge of this type of schemes can be attributed to numerous motivations, such as the need for global standardization and product traceability [5], risk management for aquaculture companies countering negative publicity [6], and as a response to inadequate regulation from public authorities [5], [7]. While they are in theory voluntary, certification schemes are becoming increasingly important to obtain access to certain markets, thus becoming a defining element of aquaculture regulation.

**Table 1:** Chosen certification schemes and standards.

Certification scheme	Standard	Version	# of indicators
Aquaculture Stewardship Council	Salmon	v1.0	152
GLOBALG.A.P.	Aquaculture/GRASP	v5.0/v1.3	267
Friend of the Sea	Marine Aquaculture	v1.1	52
International Featured Standards	IFS Food	v6.0	278
BRC Global Standards	Food Safety	v7.0	255
Royal Society for the Prevention of Cruelty to Animals	Farmed Atlantic Salmon	*09/2015*	468
Global Aquaculture Alliance/Best Aquaculture Practices	BAP Salmon	v2.3	137
Scottish Salmon Producers’ Organisation	Code of Good Practice - Seawater Lochs	*02/2015*	307

Often initiated by NGOs or retailers, certification schemes create standards and indicators of which companies need to comply to obtain the scheme׳s certification. We apply an understanding of standards and indicators which corresponds with that of the certification schemes. Hence, standards are understood as documented agreements with specific criteria that must be met in order to become certified. These standards can pertain to a specific species, a specific issue (e.g. fish health or food safety) or aquaculture in general. The criteria that make up the standards come in the form of indicators, each with corresponding requirements and guidelines for how to achieve compliance. These indicators must be measurable, transferable and comparable, allowing the same standard to be applied to a variety of local contexts.

An indicator is a measurement that can give an indication of something that is too difficult to measure in itself, such as sustainability. It is therefore not a neutral, nor a complete, representation of reality. The choice of which indicators to include in a standard, therefore, plays an important role in setting the agenda for the aquaculture industry, as it prescribes what issues are deemed important enough to address. By deciding what to count, these certification schemes are deciding what counts [8]. These choices are reflected in the data.

An important addition in this dataset is the categorization of each indicator according to topic. The list of topics was created through an iterative process between the coding of certification scheme standards and workshops with the SustainFish project׳s multidisciplinary members. This list provides a comprehensive overview of issues pertaining to sustainability of the salmon aquaculture industry, consisting of 28 topics, seven topics under each domain (economics, environment, governance, and culture). The data includes 1916 different indicators, with a total of 2830 categorizations. See Table 1 for an overview of the chosen certification schemes and standards, together with the version number of each standard and the total number of indicators for each.

## 2 Experimental design, materials and methods

The research design was based on collecting data from prevalent certification scheme standards for salmon aquaculture, and in an iterative process categorizing these standards. Through this work, we have developed a holistic, but concrete definition of sustainability applicable to the salmon aquaculture sector. A central feature of this design was to combine the expertise of scientists with insight into different scientific fields: political science, anthropology, marine social science, economics and marine biology. Furthermore, the group consists of researchers with in-depth experience with salmon aquaculture in three of the major salmon producing countries: Norway, Chile and Scotland.

The eight certification schemes were chosen based on their prevalence in Norway, Chile and Scotland. A few of the schemes are predominant in all three countries, while others are present in just one or two. While all the selected standards are applicable for salmon aquaculture companies, not all are salmon specific. Three are general aquaculture standards, while two are food safety standards. There were also certain schemes that were not included in the data, such as ISO, which was omitted because their standards are not publicly available. Access to the schemes and standards was gained through the Internet as these are publicly available in PDF format. The agency responsible for upholding the standards regularly update schemes on their website for clients and producers to see.

An initial list of topics deemed essential for making aquaculture sustainable was created through a brainstorming session with the SustainFish project members. The interdisciplinary and international character of the group allowed for comprehensive input as to what this list should include. The brainstorming session focused on topics and questions related to salmon aquaculture, which are seen to have an interaction with its surroundings both above and below water. The group was inspired by earlier work done by James [9] and others in defining sustainable cities and their criticism of the traditional 3-dimensional conceptual model of sustainability: environmental, social and economic sustainability. Their broad and holistic understanding of sustainability, which concurrently emphasizes its many specific and consequential aspects, was used as a starting point for the discussions in SustainFish. Furthermore, their approach includes competing issues and tensions, as it acknowledges that sustainability is only reachable through an assessment of conflicting priorities.

The list of topics was used to perform a preliminary coding of the *Aquaculture Stewardship Council (ASC) Salmon Standard*, through which new possible topics were discovered. The coding was done in N-VIVO. Each topic was given a separate node grouped under their respective domain (economics, environment, governance, and culture). Since many of the indicators are multifaceted, they were not restricted to one topic, but rather coded under all topics that were deemed relevant. A separate node was assigned to the indicators that did not fall under any of the chosen topics, labeled *Not Applicable*.

Based on this process, the Norwegian project members created a first version of the codebook, which was distributed to the rest of the group for feedback. Comments were then incorporated in a revised version of the codebook. This was in turn used to recode the *ASC Salmon Standard* and code seven other sustainability standards. The most recent versions of these schemes available in early spring 2017 were used in the coding (see Table 1). The version number is included in the database.

The new version of the codebook, and in particular the 273 indicators that did not fit under any topic, were presented at a second project workshop through which discussions led to a refined version. This version of the codebook consists of four domains of sustainability (economics, environment, governance, and culture) and seven subdomains (here referred to as topics) per domain. All eight standards were subsequently recoded using the new version. The list of coded indicators was then reviewed once again by the project members, divided according to the respective expertise of each researcher. Final changes were then made, based on the feedback.

**Fig. 1:**
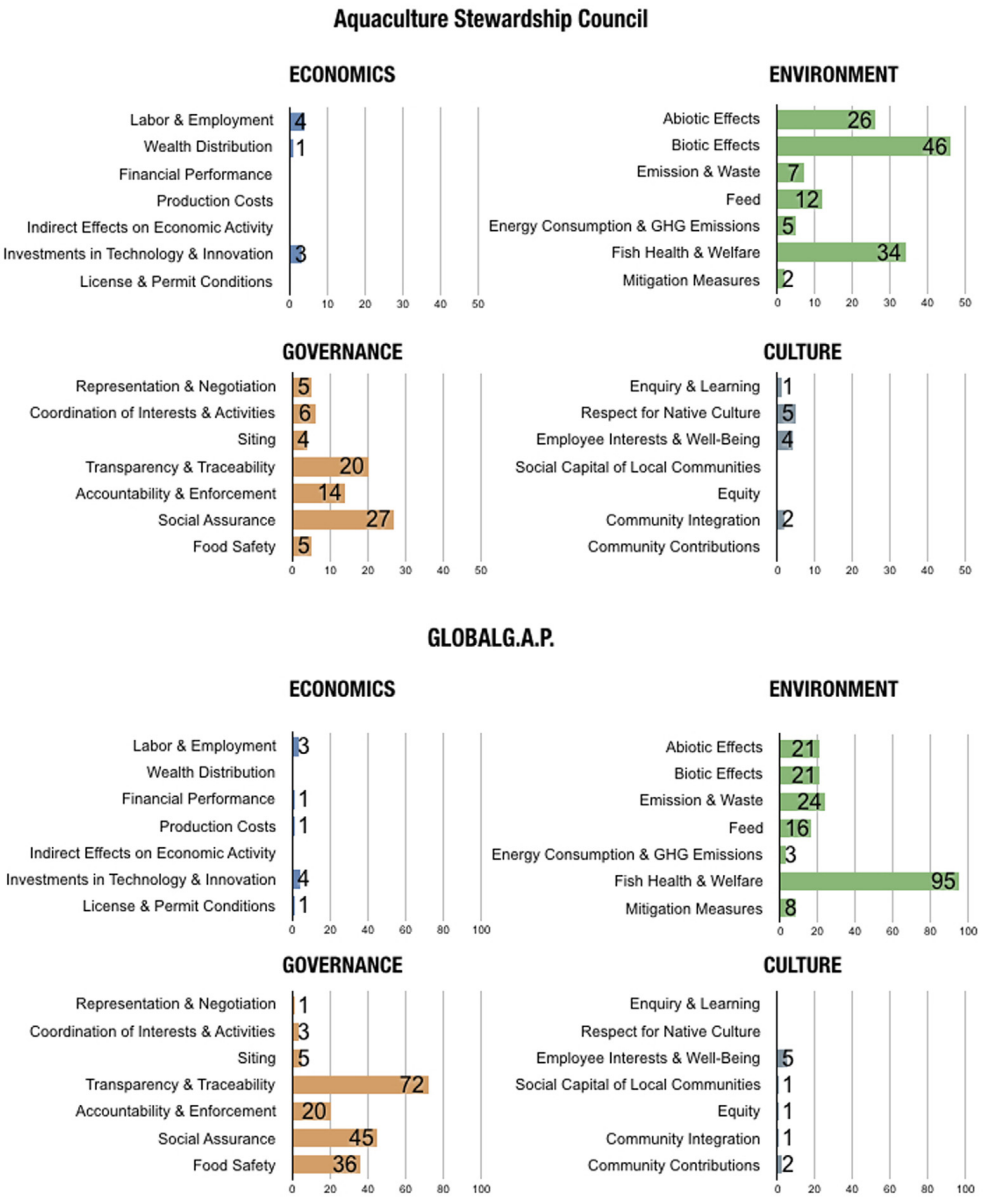

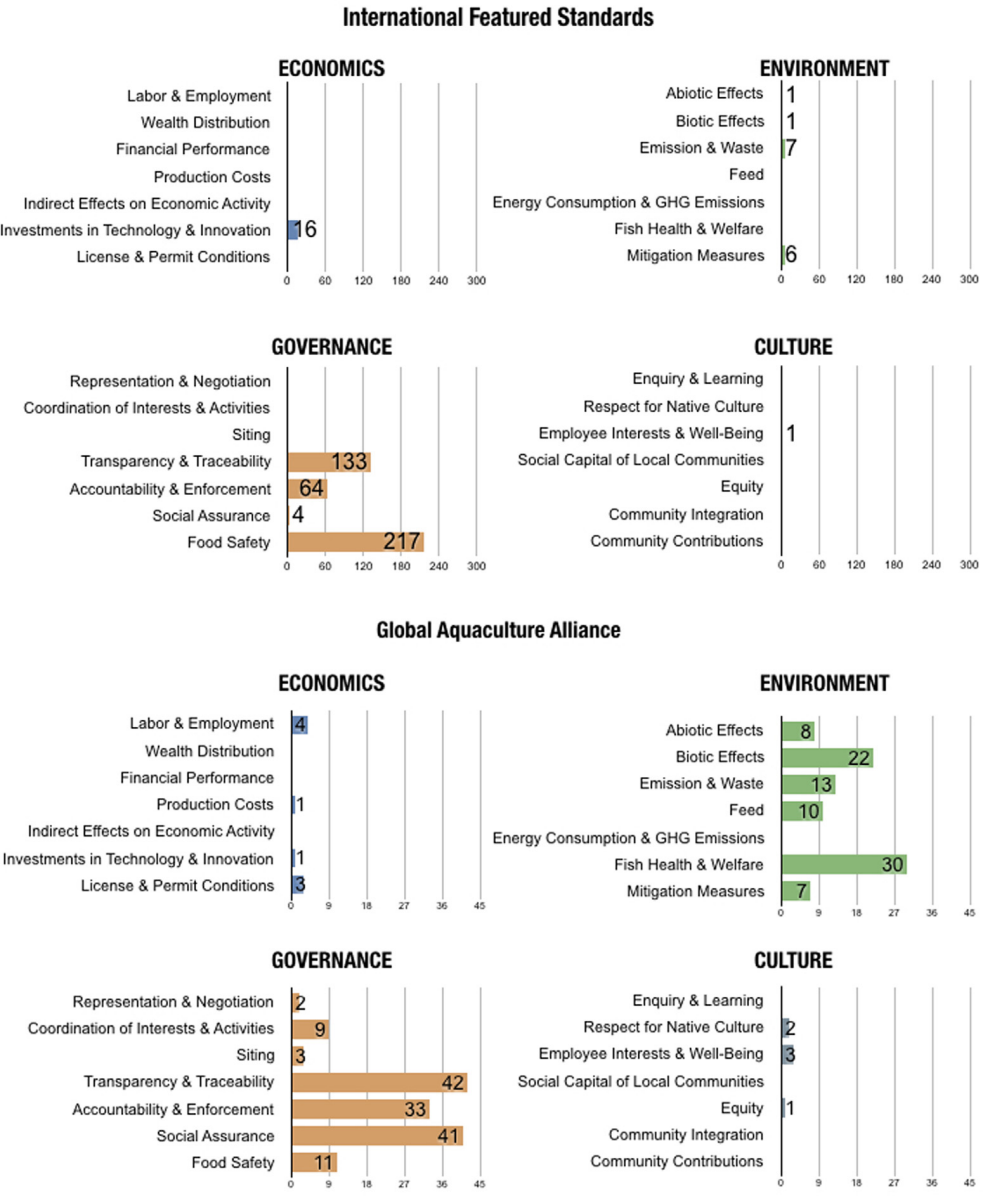

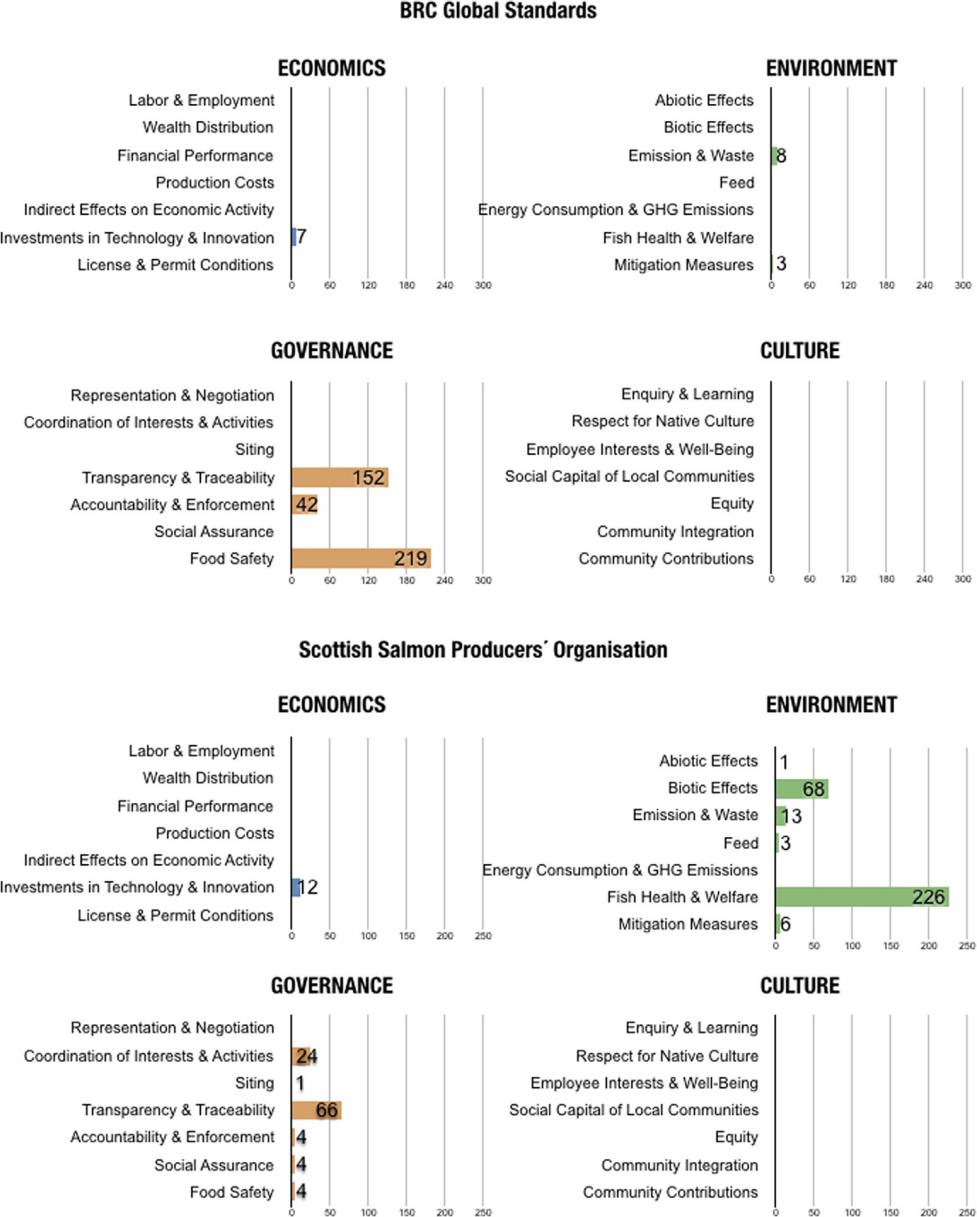

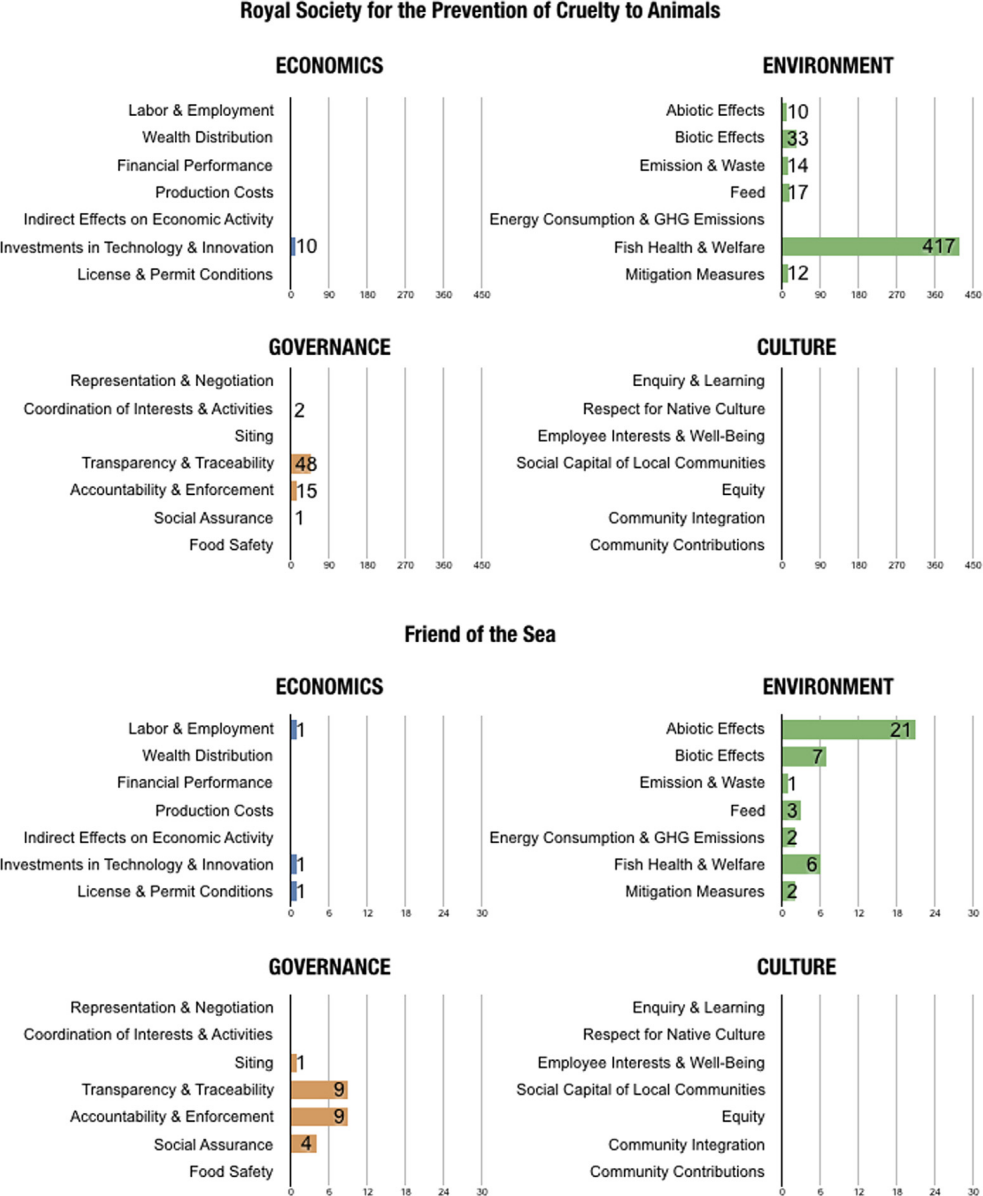
The number of indicators coded according to each topic (subdomain) for each sustainability standard.

**Fig. 2:**
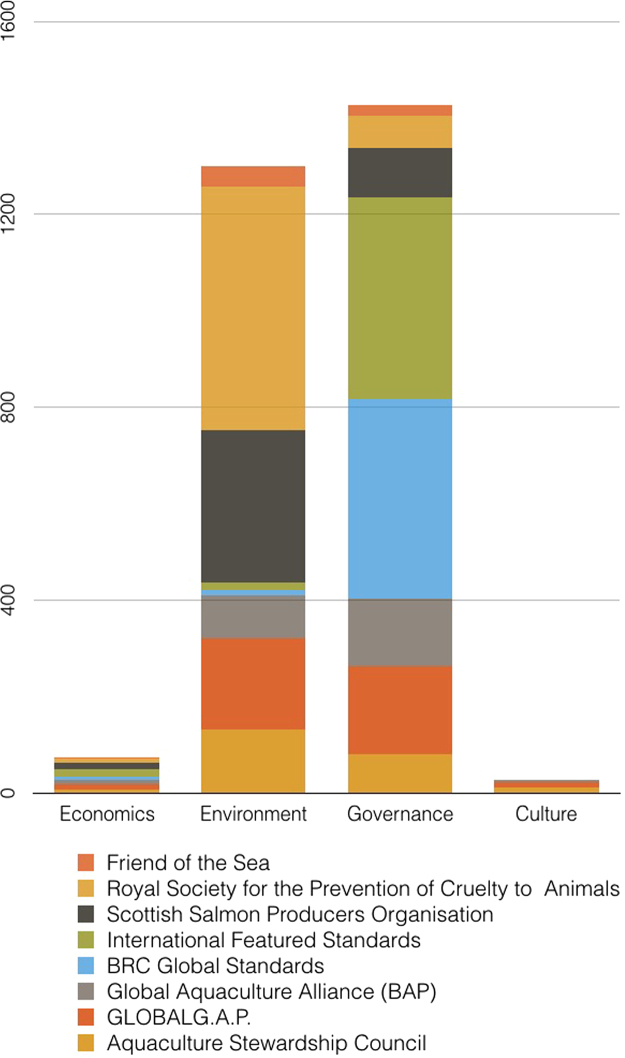
The number of indicators coded under each domain for each sustainability standard.

In the final version, no indicators were coded as *Not Applicable*. Out of all 28, there was only one topic with no relevant indicators found in the eight standards: *Indirect Effects on Economic Activities*. Table 2 shows the number of indicators coded for each topic and the corresponding sustainability standards. Fig. 1, Fig. 2 portray the coded material in two different manners, illustrating different segments and aspects of the dataset. Fig. 1 is a visual comparison of the different topics for each sustainability standard. Fig. 2 shows the content of the different standards in regards to the overarching domains.

**Table 2:** Number of indicators per topic.

	ASC	G.G.A.P	FOS	IFS	BRC	RSPCA	GAA	SSPO
ECONOMICS								
Labor & Employment	4	3	1				4	
Wealth & Distribution	1							
Financial Performance		1						
Production Costs		1					1	
Indirect Effects on Economic Activity								
Investments in Technology & Innovation	3	4	1	16	7	10	1	12
License & Permit Conditions		1	1				3	
ENVIRONMENT								
Abiotic Effects	26	21	21	1		10	8	1
Biotic Effects	46	21	7	1		33	22	68
Emission & Waste	7	24	1	7	8	14	13	13
Feed	12	16	3			17	10	3
Energy Consumption & GHG Emissions	5	3	2					
Fish Health & Welfare	34	95	6			417	30	226
Mitigation Measures	2	8	2	6	3	12	7	6
GOVERNANCE								
Representation & Negotiation	5	1					2	
Coordination of Interests & Activities	6	3				2	9	24
Siting	4	5	1				3	1
Transparency & Traceability	20	72	9	133	152	48	42	66
Accountability & Enforcement	14	20	9	64	42	15	33	4
Social Assurance	27	45	4	4		1	41	4
Food Safety	5	36		217	219		11	4
CULTURE								
Enquiry & Learning	1							
Respect for Native Culture	5						2	
Employee Interests & Well-Being	4	5		1			3	
Social Capital for Local Communities		1						
Equity		1					1	
Community Integration	2	1						
Community Contributions		2
